# Intraindividual variance of lower limb rotation in patients with bilateral knee osteoarthritis

**DOI:** 10.3389/fsurg.2023.964160

**Published:** 2023-03-01

**Authors:** Xin Zheng, Yang-yu-fan Wang, Wang-yi Jin, Chao-ran Huang, Zi-wen Yan, Da-lin Peng, Shen Zhou, Kai-jin Guo, Sheng Pan

**Affiliations:** ^1^ Department of Orthopaedics, Zhujiang Hospital, Southern Medical University, Guangzhou, China; ^2^ State Key Laboratory of Pharmaceutical Biotechnology, Department of Sports Medicine and Adult Reconstructive Surgery, Nanjing Drum Tower Hospital, The Affiliated Hospital of Nanjing University Medical School, Nanjing, China; ^3^ Department of Traumatic Surgery, Changshu Hospital Affiliated to Soochow University, First Peoples' Hospital of Changshu, Changshu, China; ^4^ Department of Orthopaedics, The Affiliated Hospital of Xuzhou Medical University, Xuzhou, China; ^5^ Department of Orthopedics, The Second Affiliated Hospital of Soochow University, Suzhou, China

**Keywords:** external, rotation, osteoarthritis, computed tomography, individual

## Abstract

**Purpose:**

To determine the side-to-side difference in intraindividual rotation alignment of patients with bilateral varus-type knee osteoarthritis (OA) and compare it with control subjects.

**Methods:**

This retrospective study enrolled 60 patients with bilateral varus-type knee OA and 50 control subjects. All cases underwent bilateral lower limb CT angiography. Bilateral femoral and tibial rotation alignment were measured, and the overall lower limb rotation was calculated by two different methods. Method 1 was calculated by subtracting angle of the femoral torsion from the tibial torsion and method 2 was determined by relative rotation of the femoral neck angle to bimalleolar angle. The intraindividual variance and differences between the two groups were analyzed.

**Results:**

Both OA and control samples showed significant differences between right and left for all measurements. Femoral torsion for control group was 10.4 ± 5.5°, tibial torsion was −22.1 ± 6.1°, and overall leg rotation by method 1 was −15.6 ± 7.2° and method 2 was −11.7 ± 8.2°. Femoral torsion, tibial torsion, method 1, and method 2 in the patients with OA were 8.2 ± 6.3°, −18.6 ± 4.1°, −14.9 ± 7.9°, and −10.4 ± 7.6°, respectively. Patients with OA showed a more pronounced retroversion in the femur (*p *= 0.008) and more internal rotation in the tibia (*p *<* *0.001). No statistical significance of both methods was found between the two groups. Patients with OA had a greater median side-to-side absolute difference in all measurements, though the differences of both two methods of overall lower limb rotation were not statistically significant.

**Conclusions:**

The discrepancy of side-to-side differences of bilateral lower limb rotation ought to be noticed with caution in diagnosing and treating rotational deformities of the lower limb, especially for patients with bilateral knee OA.

## Background

Improper rotational alignment of the lower limb may be carried over from childhood or acquired (1). Open or closed reduction and intramedullary nailing of femur and tibia fractures is a long-standing and less-invasive surgical procedure (2). Fracture commonly achieves indirect healing for the stable fixation of closed nailing, whereas the reconstruction of anatomical rotational alignment of the lower limb is of vital importance (3, 4). Contralateral healthy knee was always used as reference of rotation for reconstruction of the pre-traumatic alignment. Rotational differences of over 15°, compared to the healthy side, are considered to be unacceptable (3, 5, 6).

Computed tomography (CT) is the current gold standard for the measurements of axial rotational alignment (7). The rotational alignment is composed of four axes of femur and tibia: femoral neck axis, distal femoral condylar axis, the proximal tibial axis, and bimalleolar axis. “Ulm method” was one of the most widely accepted techniques for measuring femoral, tibial, and limb rotation described by Waidelich et al. (2). In 2011, Liodakis et al. (8) proposed an alternative method measuring the overall lower limb rotation (neck–malleolar angle) that considers the knee joint rotation angle.

It was reported that there is a significant side-to-side difference of bilateral femorotibial torsion in healthy subjects (1). Knee osteoarthritis (OA) is one of the most common forms of OA, especially in people over 50 years old. Patients with knee OA tend to combine with a deformity of the lower limbs (9). In a study of the strength of the associations of knee injury and obesity with OA in 3,885 healthy people, it was found that the incidence of bilateral osteoarthritis is 5%, which is more common than unilateral osteoarthritis (2%) (10). Günther et al. (11) also reported that in individuals who have primarily unilateral knee OA, 87.4% had radiographic evidence of bilateral OA. There was less femoral anteversion and more external torsion of tibia in patients with knee osteoarthritis than normal subjects (9, 12, 13). However, to date, there is a distinct lack of literature characterizing the side-to-side variations in rotation of the lower limbs in patients with bilateral knee OA.

The purpose of this study was to determine the side difference in intraindividual rotation alignment of patients with bilateral knee OA and to compare it to control subjects. The hypothesis was that patients with bilateral knee OA had a greater mean side-to-side absolute difference than control subjects. The finding may be beneficial to acute clinical settings as well as for orthopedist opinion.

## Materials and methods

The study was approved by the hospital review board, and each patient enrolled was given a written informed consent. All cases who underwent bilateral lower limb CT angiography from January 2018 to December 2020 in our hospital were identified using the hospital's Picture Archiving and Communication System (PACS; GE Healthcare, Chicago, IL, United States) for vascular disease of lower limb. In this retrospective study, all images were judged by two experienced orthopedic surgeons and the diagnosis of OA was done *via* coronal reconstruction of CT or standard x-rays of the knee joints. Sixty patients with bilateral varus-type knee OA and 50 patients who were not diagnosed with OA but were suspected of having other lower limb diseases such as deep venous thrombosis or arterial embolism were enrolled.

Lower limb CT angiographies were done with Philips iCT256 (Philips, Netherlands). Patients were positioned in a supine state of neutral rotation with knees fully extended, feet affixed, and toes pointing upward. Scan level ranged from the ilium to the distal of the feet, including the joints of the hip, knee, and ankle with sections of 0.625 mm thickness. The image was obtained with radiation levels of 100–120 kVp for an effective mAs (20–35 mAs) duration.

For the OA group, patients were exclude if they had (1) a diagnosis other than primary knee OA, (2) the Kellgren–Lawrence (K–L) grade of either side of knee was lower than 2, (3) a significant bony deformity that restrained identification of the anatomical landmarks for measurement, (4) a history of operation on the lower limb (e.g., total hip arthroplasty, an operation for a femoral or tibial fracture, or high tibial osteotomy), and (5) amputation of the calf/thigh.

The exclusion criteria of the control subjects were (1) age younger than 18 years, (2) osteoarthritis of the hip and knee joint, (3) endoprosthesis of the hip or knee joint, (4) post-traumatic changes of the lower leg (e.g., acute fracture), (5) bony abnormalities (e.g., tumors or severe deformities), and (6) amputation of the lower leg.

All data were measured on PACS by two independent observers who were familiar with the rotational assessment of the lower limbs on the axial plane of CT images in two times with each at a 1-month interval. Four axes were measured in the lower limb: femoral neck axis, posterior condylar axis (PCA) of the distal femur, axis of the proximal tibial condyles, and bimalleolar axis. The data were recorded as an angle between the axis and the horizontal plane on PACS. Internal rotation was assigned a negative sign, and external rotation was assigned a positive sign. The angles were measured to the least 0.1°.

**Figure 1:**
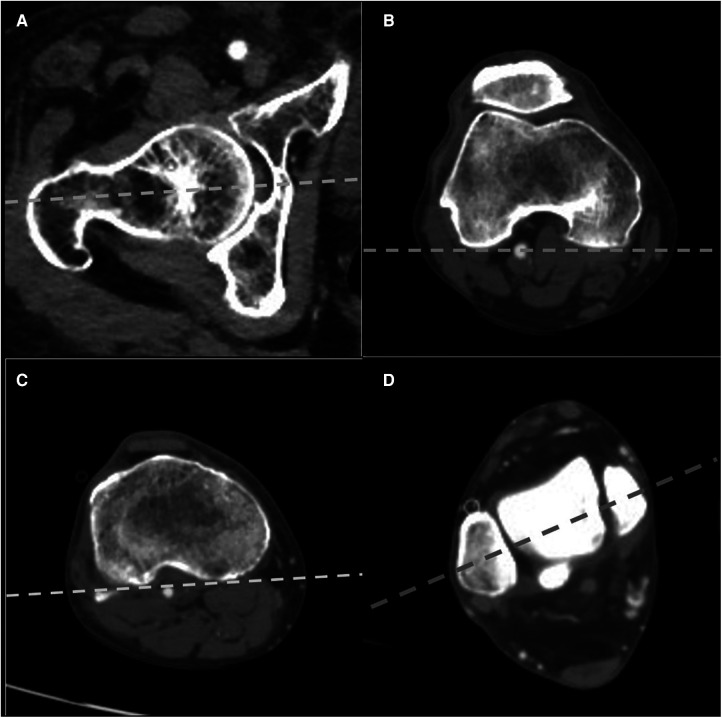
Measurement technique of lower limb rotation on CT (right limb). (**A**) Femoral neck axis. (**B**) Posterior condylar axis of the distal femur. (**C**) Axis of the proximal tibial condyles. (**D**) The bimalleolar axis. The data were recorded as an angle between the axis and horizontal baseline. CT, computed tomography.

The femoral neck axis was defined as a line connecting the center of the femoral head and the midpoint of the femoral neck with the femoral head, isthmus of the femoral neck, and the superior border of the greater trochanter is evident in a CT cut (Figure 1A). This method was first proposed by Hernandez et al. (14) and validated by Liodakis et al. (8). The PCA of the distal femur was defined as a line connecting the posterior margins of the lateral and medial femoral condyles (9) (Figure 1B). The axis of the proximal tibial condyles was defined as a line between posterior cortices of the proximal tibial condyles, set at the plane of the apex of the fibula (1). (Figure 1C) The bimalleolar axis was defined as a line connecting the centers of the medial and lateral malleolus (6) (Figure 1D).

The following step was to calculate the rotational profile of lower limb. The femoral torsion was calculated by subtracting the posterior condylar axis from the femoral neck angle (1). Femoral anteversion and retroversion were represented by positive values and negative values, respectively. Femoral anteversion was assigned femoral external rotation of the femoral neck in relation to the PCA.

The tibial rotation was calculated by subtracting the bimalleolar angle from the angle of the proximal tibial condyles (1). Positive values indicated the internal rotation of the tibia, and negative values indicated the external rotation of the tibia. Negative values presented external rotation of baseline of the proximal posterior tibia in relation to the distal tibia.

The overall lower limb rotation had two methods as reported: method 1 was calculated by subtracting angle of the femoral torsion from the tibial torsion; method 2 was reported as the neck–malleolar angle proposed by Liodakis et al. (8) in 2011 and determined by relative rotation of the femoral neck angle to bimalleolar angle. The angle was calculated by subtracting the bimalleolar angle from the femoral neck angle. Negative values represented relative external rotation of distal tibia in relation to the proximal femur.

### Statistical analysis

For the OA group, an estimated sample size of at least 34 would be needed to provide 80% power for two-sided paired sample t tests, assuming an effect size index of 0.5, with a two-sided *α* of 0.05. Statistical analysis was performed with SPSS 25.0 (IBM, Chicago, Illinois, United States). All data were obtained by two independent observers in two times with each at a 1-month interval. The mean value of the four different measurements was used for analysis. Inter- and intraobserver reliabilities of the methods were evaluated by intraclass correlation coefficients (ICCs). ICC values greater than 0.80 indicated excellent reliability. The normality of continuous variable was calculated by the Shapiro–Wilk test. Data with normal distribution were expressed as mean and SD. Difference of variables was reported as absolute value. All variables but the side-to-side absolute difference of the lower limb rotation (both patients with OA and control subjects) were normally distributed. Two-sided paired t test was conducted to determine significant differences between right and left limb for all measurements. Independent-sample T tests or Mann–Whitney *U* test was conducted to determine the differences of the two groups or two methods of overall lower limb rotation. *p *<* *0.05 was considered as statistically significant.

## Results

There were no significant differences between the OA and control groups on gender (*p *= 0.275) and age (*p *= 0.208).

For all parameters, ICCs of inter- and intraobserver reliabilities were all greater than 0.90, which indicated excellent reliability.

Both OA and control samples showed significant differences between right and left for all measurements (Table 1).

**Table 1:** Results of bilateral measurements separately shown for OA and control subjects.

	OA group (*n* = 60)			Control group (*n* = 50)		
	Left mean ° (SD)	Right mean ° (SD)	*p-*value	Left mean ° (SD)	Right mean ° (SD)	*p-*value
Femur rotation	9.0 (6.8)	7.5 (5.7)	0.003*	9.7 (5.8)	11.2 (5.3)	0.001*
Tibial rotation	−17.6 (4.1)	−19.6 (3.9)	0.001*	−23.0 (7.2)	−21.4 (4.0)	0.009*
Method 1	−13.0 (8.1)	−16.7 (7.3)	0.001*	−16.7 (8.1)	−14.6 (6.1)	0.002*
Method 2	−8.6 (7.9)	−12.2 (6.9)	0.001*	−13.3 (9.3)	−10.2 (6.9)	0.001*

OA, osteoarthritis.

* p < 0.05 [Normally distributed values were given as means and SD].

Femoral torsion, tibial torsion and overall leg rotation by the two methods of the control group were provided in Table 2.

**Table 2:** Differences of measurement between OA and control subjects.

		OA (°)	Control (°)	*p-*value
Femur rotation	Overall	8.2 (6.3)	10.4 (5.5)	0.008*
	AbsΔ (right–left)	2.7 (1.4–5.1)	1.7 (1.0–2.8)	0.010*
Tibial rotation	Overall	−18.6 (4.1)	−22.1 (6.1)	0.001*
	AbsΔ (right–left)	3.9 (2.7–5.4)	2.6 (1.1–5.6)	0.030*
Method 1	Overall	−14.9 (7.9)	−15.6 (7.2)	0.490
	AbsΔ (right–left)	4.7 (3.2–6.7)	3.7 (2.1–5.4)	0.040*
Method 2	Overall	−10.4 (7.6)	−11.7 (8.2)	0.217
	AbsΔ (right–left)	5.4 (3.4–7.7)	4.7 (2.1–6.7)	0.085

OA, osteoarthritis; AbsΔ, absolute difference.

* p < 0.05 [Normally distributed values were given as means and SD; Non-normal variables were reported as median (interquartile range)].

Femoral and tibial torsion in the patients with OA were 8.2 ± 6.3° and −18.6 ± 4.1°, respectively. The overall lower limb rotation calculated by method 1 and method 2 were 14.9 ± 7.9° and −10.4 ± 7.6°, respectively (Table 2). Patients with OA showed a more pronounced retroversion in the femur (*p *= 0.008) and more internal rotation in the tibia (*p *<* *0.001). No statistical significance of both methods was found between bilateral varus-type knee OA group and control group (Table 2).

**Figure 2:**
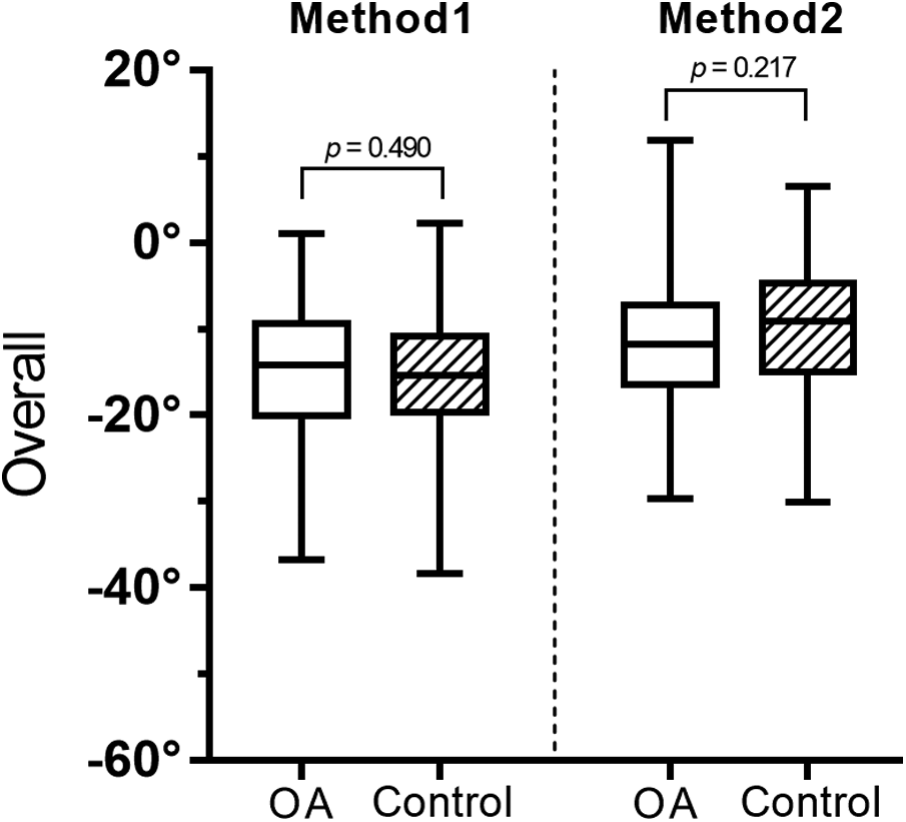
Absolute difference between methods 1 and 2 by a box-whisker plot. OA, osteoarthritis; AbsΔ, absolute difference.

Patients with OA had a more evident median side-to-side absolute difference in all measurements, though the differences of two methods of overall lower limb rotation were not statistically significant (Table 2 and Figure 2).

**Figure 3:**
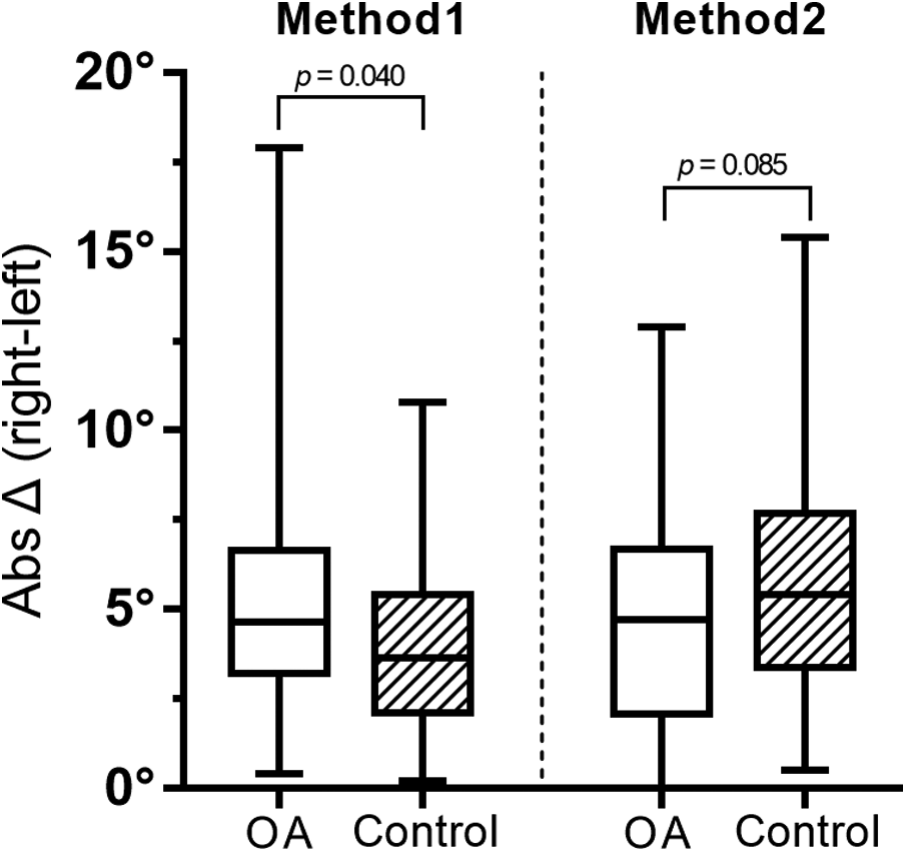
Overall results of methods 1 and 2 by a box-whisker plot. OA, osteoarthritis.

For both OA and control groups, there were no significant differences between the two methods in terms of side-to-side absolute difference of overall lower limb rotation (Figure 3).

## Discussion

This study aimed to investigate the side-to-side difference in intraindividual rotation alignment of patients with bilateral knee OA and to compare it to control subjects. The key findings of this study including the following: First, there were significant differences between right and left for all measurements of lower limb rotation in both OA group and control subjects. Second, patients with bilateral knee OA had a more evident side-to-side difference in all measurements compared with control subjects. It may be profitable to acute clinical settings or subsequent orthopedic procedures, especially for those patients with bilateral knee OA.

With regard to the reduction and fixation for fractures, the reconstruction of long bone of the lower limb into a correct rotational limb alignment is necessary (3). The healthy side is usually used as the reference to restore pre-traumatic lower limb rotation alignment (2). Side-to-side difference in the lower limb rotation was described by numerous studies (1, 15). Strecker et al. (15) analyzed the intraindividual asymmetry of lower limb rotation in 355 normal individuals. The rotation of right and left femur in individuals did not differ significantly, but there was a significant difference of rotation between right (36.46° of external torsion) and left tibia (33.07° of external torsion). It was reported that two methods identifying the overall lower limb rotation to determine the intraindividual variance of bilateral lower limb rotation in 105 healthy subjects and showed the mean side-to-side differences of 6.0 ± 4.7° in femoral and 5.7 ± 4.8° in tibial rotation. The absolute side-to-side overall lower limb rotation difference was 9.5° with both methods. Our study found the conclusion of the intraindividual side difference in bilateral “healthy” legs, though the median absolute difference of lower limb rotation was less than previous studies (1).

Traditional method measuring the overall lower limb rotation is calculated from the differences in the femoral and tibial rotation (2). This method was regarded negligence of the variability of knee joint rotation. It is known that the magnitude of rotational shift during knee extension relies on the individual difference of the medial and lateral femoral condyles (16). The internal and external rotation of the tibia relative to the femur is minimal at full extension of the knee. Consequently, it is evident that the knee joint rotation is an important anatomical element of the overall lower limb rotation. Liodakis et al. (8) were the first to introduce an alternative method (neck–malleolar angle) for measuring overall lower limb torsion that considers the knee joint rotation angle. This method was a direct measurement of the angle between the femoral neck axis and the bimalleolar axis. They used both the traditional method and the method of neck–malleolar angle to determine the overall lower limb rotation; however, the difference between the two methods was not mentioned. Overall leg rotations calculated by two methods were compared with the study by Ries et al. (1). The absolute side-to-side differences of overall lower limb rotation by two methods were both 9.5°. The differences between both methods were not significant. Yet, absolute differences between the two methods were 3.3°. In our study, for both OA and control subjects, no significant differences between the two methods in terms of side-to-side absolute difference of overall lower limb rotation was found.

Khan et al. (17) noticed that tibial rotation reduced significantly in OA patients (19.5 ± 6.16°) compared with that in the healthy group (23.51 ± 6.34°). They also reported a significant negative correlation between varus deformity and tibial torsion (*r* = −0.54, *p *<* *0.02). It was indicated that as the progression of varus deformity, tibial torsion reduced further. Chang et al. (9) divided 422 lower limbs into three groups according to the coronal alignment. In their study, as the coronal alignment changed from varus to valgus, the degree of femoral anteversion (the angle between femoral neck and PCA) and the external tibial rotation increased. However, their external tibia rotation was determined by the angle between PCA and the line connecting the most prominent points of lateral and medial malleolus. According to the study by Liodakis et al. (6), the bimalleolar methods used in our study for measuring rotation had the greatest inter- and intraobserver reliabilities. In the current study, we compared the mean rotation alignment of bilateral lower limb of OA patients with control subjects. Significant differences were found both in the femoral and tibial rotation between the two groups, but the overall lower limb rotation by two methods was not significantly different. Previous reports of lower limb rotation were in agreement with our findings, namely, those of Moussa (18), who evaluated the difference of rotational alignment patterns between OA patients and control subjects who had no knee joint problems. Moreover, in our study, all differences of bilateral lower limbs were reported as absolute values. In our view, there was no need to determine which side rotates more internally or externally.

There were several limitations of this study. First, patients with OA always have different degrees of articular cartilage erosion, which is invisible using CT. In addition, the behavior of the ligament apparatus is also hard to be determined by CT. It may be considered to recommend magnetic resonance imaging (MRI) in future studies. Second, most cases underwent lower limb CT angiography for thrombosis of lower limbs. Those were unable to represent the general normal population. Also, age-related changes in bone morphology for control subjects, therefore, cannot be ruled out. However, due to ethical considerations, we could not acquire lower limb CT angiography of healthy subjects. Finally, it was a retrospective study, the knee OA was only diagnosed by K–L grades on the coronal plane of CT reconstruction or standard short knee radiograph. We could not obtain the patients’ specific clinical symptoms (e.g., pain intensity and knee function). Furthermore, we were unable to ensure the same bilateral K–L grades of knees in patients with bilateral knee OA. Further studies may consider the association between a larger sample size of symptomatic knee OA and control samples concerning lower limb rotation.

## Conclusion

Compared with control subjects, patients with bilateral knee OA had a greater side-to-side absolute difference in all measurements. The discrepancy ought to be noticed with caution in diagnosing and treating rotational deformities of the lower limb, especially for patients with bilateral knee OA.

## Data Availability

The raw data supporting the conclusions of this article will be made available by the authors, without undue reservation.
